# Supplementary material for: A Humanized Yeast Phenomic Model of Deoxycytidine Kinase to Predict Genetic Buffering of Nucleoside Analog Cytotoxicity
Source: Genes (Basel). 2019 Sep 30;10(10):770. doi: 10.3390/genes10100770 (PMC6826991; doi:10.3390/genes10100770)
Supplement: Supplementary file 1 [file genes-10-00770-s001.zip › Additional_Files/Additional_File7_GOTermHeatmaps.zip!/Additional_File7_GOTermHeatmaps/endonucleolytic_cleavage_in_ITS1_to_separate_SSU-rRNA_from_5.8S_rRNA_and_LSU-rRNA_from_tricistronic_rRNA_transcript_(SSU-rRNA,_5.8S_rRNA,_LSU-rRNA).pdf]

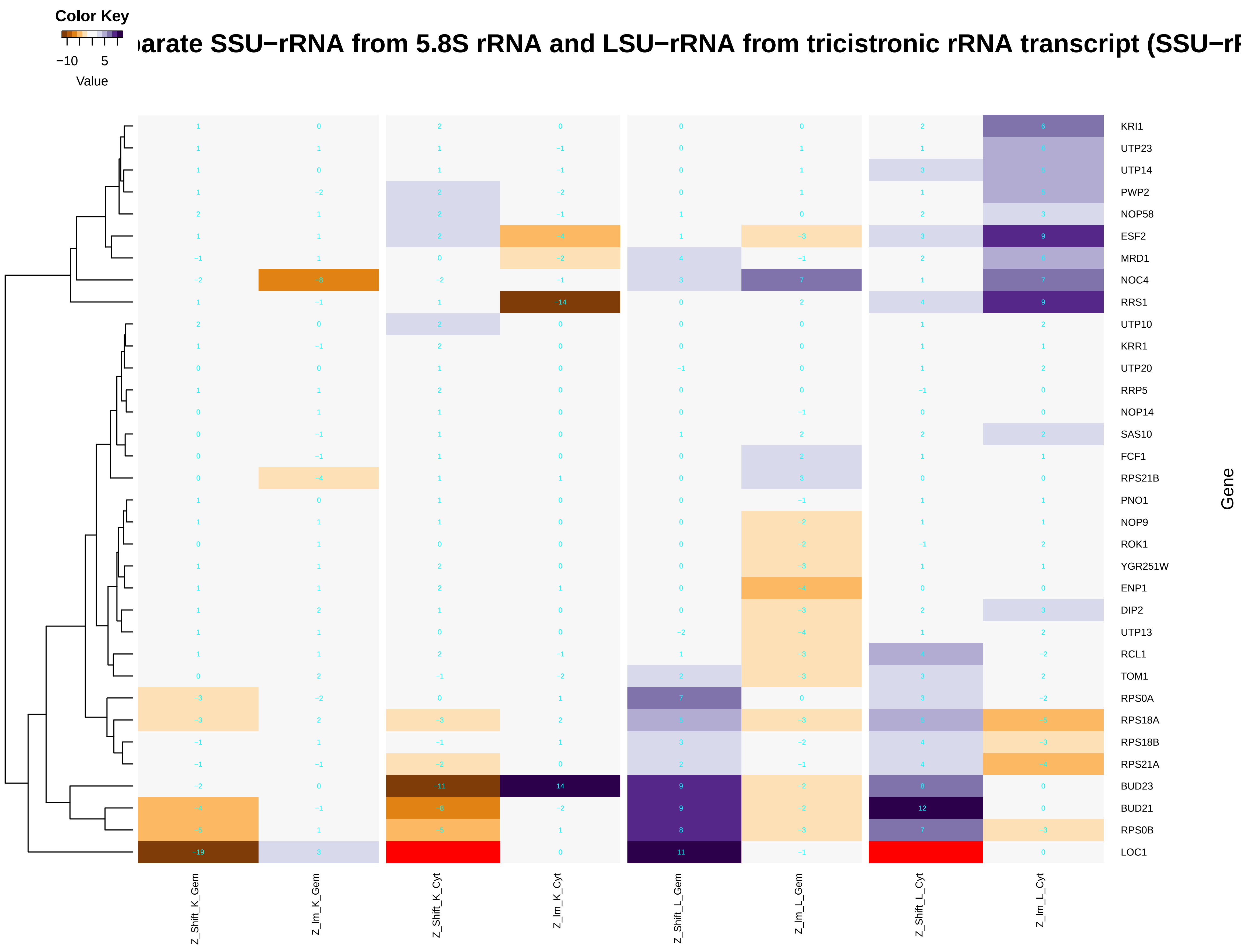

Color Key
−10 5
Value
ıarate SSU−rRNA from 5.8S rRNA and LSU−rRNA from tricistronic rRNA transcript (SSU−rF
KRI1
UTP23
UTP14
PWP2
NOP58
ESF2
MRD1
NOC4
RRS1
UTP10
KRR1
UTP20
RRP5
NOP14
SAS10
FCF1
RPS21B
PNO1
NOP9
ROK1
YGR251W
ENP1
DIP2
UTP13
RCL1
TOM1
RPS0A
RPS18A
RPS18B
RPS21A
BUD23
BUD21
RPS0B
LOC1
Gene
Z_Shift_K_Gem
Z_Im_K_Gem
Z_Shift_K_Cyt
Z_Im_K_Cyt
Z_Shift_L_Gem
Z_Im_L_Gem
Z_Shift_L_Cyt
Z_Im_L_Cyt
